# Epidemiology of Nontuberculous Mycobacterial Infection, South Korea, 2007–2016

**DOI:** 10.3201/eid2503.181597

**Published:** 2019-03

**Authors:** Hyewon Lee, Woojae Myung, Won-Jung Koh, Seong Mi Moon, Byung Woo Jhun

**Affiliations:** Seoul National University, Seoul, South Korea (H. Lee);; Seoul National University Bundang Hospital, Seoul (H. Lee, W. Myung);; Sungkyunkwan University School of Medicine, Seoul (W.-J. Koh, S.M. Moon, B.W. Jhun)

**Keywords:** nontuberculous mycobacteria, epidemiology, bacteria, mycobacteria, South Korea, tuberculosis and other mycobacteria

## Abstract

The prevalence and incidence of nontuberculous mycobacterial (NTM) infections increased in South Korea from 2007 to 2016. Annual prevalence of NTM infection increased to 39.6 cases/100,000 population in 2016 and annual incidence to 19.0 cases/100,000 population. Overall prevalence for the study period was higher in the elderly, in females, and in cities.

The prevalence and incidence of nontuberculous mycobacterial (NTM) infection are increasing worldwide (*1*), and it is important to characterize the distribution of NTM infection by demographics or ecologic region to optimize disease control. However, data vary widely by study, and the epidemiology of NTM infection differs among countries. Although the number of patients with NTM infection has increased in South Korea (*2*), representative nationwide population-based epidemiologic data on the extent and distribution of NTM infection are lacking. We evaluated the prevalence, incidence, and spatial distribution of NTM infection in South Korea over a 10-year period, using national health insurance data.

## The Study

We based our study on the Health Insurance Review and Assessment Service database, which includes universal health insurance claim data in South Korea. Approximately 97% of the population (≈52 million persons) is included in the system (*3*). We obtained demographic and medical data from the database for 2007–2016. We defined NTM infection as the presence of a diagnostic code associated with NTM infection (International Classification of Diseases, 10th ed., code A31) as either a primary or secondary diagnosis (*4*) and claims data for acid-fast bacilli smears or mycobacterial culture. 

We defined annual prevalence as the number of all patients with NTM infection alive during each year and annual incidence as the number of newly diagnosed NTM infections occurring each year after excluding patients who were diagnosed in a previous study period (e.g., patients diagnosed during 2007–2015 were excluded from the estimate of the 2016 incidence rate). We first calculated the crude prevalence rate and incidence rate using each year’s total population as the denominator. To adjust for different population structures between years, we calculated the age- and sex-standardized annual prevalence rate and incidence rate of NTM disease using the 2005 population as a standard population, based on the Statistics Korea method (http://kostat.go.kr/portal/eng/index.action). First, we calculated the prevalence and incidence rates for age and sex groups by stratifying all NTM cases into the groups and dividing by the group-specific populations in each year. Then, we multiplied each year’s group-specific rates and the group-specific populations in 2005, combined the multiplied values, and divided by the 2005 total population. We calculated standardized overall period prevalence of NTM infection by age, sex, and administrative divisions using the population in the middle of the study period. We conducted statistical analyses using SAS version 9.4 (SAS Institute, http://www.sas.com). Because we used deidentified data in the study, institutional review board approval and patient consent were not required.

**Figure 1:**
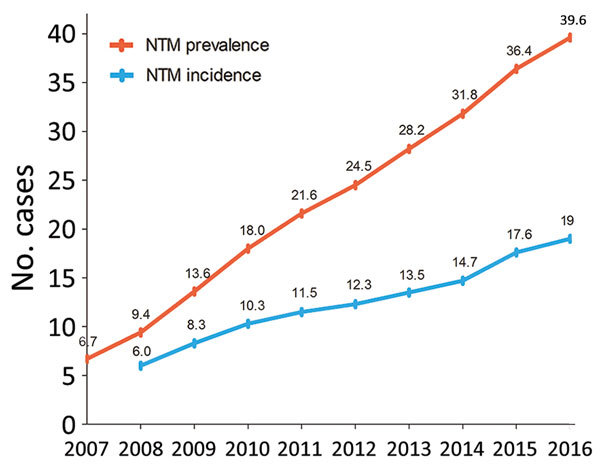
Annual prevalence (total no. cases/100,000 population) and incidence (no. new cases/100,000/year) of nontuberculous mycobacterial infection, adjusted for age and sex, South Korea, 2007–2016.

**Figure 2:**
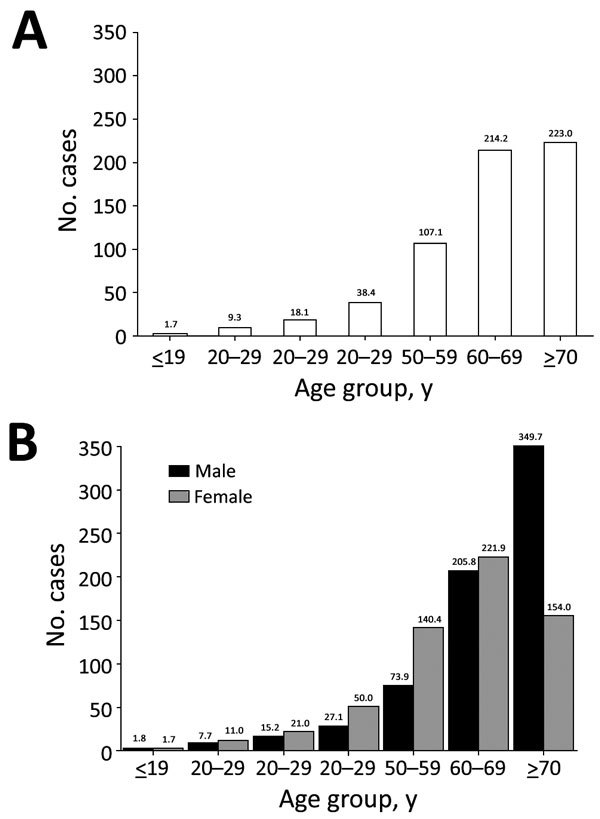
Overall period prevalence (total no. cases/100,000 population) of nontuberculous mycobacterial infection, by age group (A) and by age group and sex (B), South Korea, 2007–2016.

A total of 33,974 cases of NTM infection were identified during 2007–2016. The age- and sex-adjusted annual prevalence of NTM infection increased by more than 5-fold, from 6.7 cases/100,000 population in 2007 to 39.6 cases/100,000 population in 2016 (Figure 1). The age- and sex-adjusted annual incidence of NTM infection more than tripled, from 6.0 cases/100,000 population/year in 2008 to 19.0 cases/100,000 population/year in 2016. The overall period prevalence of NTM infection increased with patient age (Figure 2, panel A): prevalence was lowest at <19 years of age (1.7 cases/100,000 population), increased dramatically after 50 years of age, and peaked in those *>*70 years of age (223.0 cases/100,000 population). The overall period prevalence of NTM infection was higher among females in all age groups, except for those >70 years of age (Figure 2, panel B).

In South Korea, the overall period prevalence of NTM infection tended to be higher in metropolitan cities, which had greater population densities and higher income levels, than in the provinces (Table 1). The overall period prevalence exceeded 100 cases/100,000 population in most metropolitan cities (5/7, 71.4%) but did not exceed that rate in most provinces (7/9, 77.8%). The prevalence was highest in Gwangju and in Seoul, the capital of South Korea, both of which had >250 cases/100,000 population. Prevalence was lowest in Cheju, the largest and southernmost island (35.8 cases/100,000 population) (Appendix Figure).

**Table 1:** Overall prevalence of nontuberculous mycobacterial infections by administrative division, adjusted for age and sex, South Korea, 2007–2016

District	No. infections	No. cases/100,000 population
Metropolitan city		
Seoul	13,671	254.5
Busan	2,055	102.6
Daejeon	1,384	194.4
Gwangju	2,121	302.0
Daegu	1,642	119.9
Incheon	1,169	86.3
Ulsan	401	81.5
Province		
Gyeonggi-do	6,195	110.2
Gangwon-do	1,107	108.9
Jeollabuk-do	1,213	98.4
Jeollanam-do	470	35.1
Gyeongsangbuk-do	497	26.8
Gyeongsangnam-do	1,201	66.0
Chungcheongbuk-do	285	30.6
Chungcheongnam-do	444	35.2
Cheju-do (island)	119	35.8

Analysis of age and underlying conditions indicated that most case-patients (83.4%) were >50 years of age (Table 2). The most frequent underlying respiratory condition was asthma (53.0%), followed by bronchiectasis (43.6%) and chronic obstructive pulmonary disease (COPD) (32.3%). Lung cancer was the most common malignancy (6.3%). Depressive disorder was noted in 23.1% of case-patients.

**Table 2:** Sex, age, and underlying conditions among patients with nontuberculous mycobacterial infection, South Korea, 2007–2016*

Category	No. (%) infections, n = 33,974
Sex	
F	18,555 (54.6)
M	15,419 (45.4)
Age group, y	
<19	197 (0.6)
20–29	622 (1.8)
30–39	1,479 (4.4)
40–49	3,341 (9.8)
50–59	7,880 (23.2)
60–69	8,961 (26.4)
>70	11,494 (33.8)
Underlying condition	
Asthma	18,003 (53.0)
Bronchiectasis	14,815 (43.6)
COPD	10,969 (32.3)
Interstitial pulmonary diseases	1,832 (5.4)
Myocardial infarction	1,016 (3.0)
Congestive heart failure	3,581 (10.5)
Cerebrovascular disease	7,789 (22.9)
Cancer	7,808 (23.0)
Lung cancer	2,124 (6.3)
Stomach cancer	1,336 (3.9)
Colon cancer	975 (2.9)
Liver cancer	661 (2.0)
Prostate cancer	606 (1.8)
Breast cancer	577 (1.7)
Uterine cervical cancer	126 (0.4)
Thyroid cancer	673 (2.0)
Liver disease	15,218 (44.8)
Renal disease	1,391 (4.1)
Diabetes mellitus	11,010 (32.4)
Depressive disorder	7,857 (23.1)

*Underlying conditions are listed based on codes from the International Classification of Diseases, 10th edition. COPD, chronic obstructive pulmonary disease.

## Conclusions

We identified a remarkable increase in the prevalence and incidence of NTM infection in South Korea from 2007 to 2016. The overall period prevalence increased with patient age and was higher in female patients in most age groups. These trends are consistent with previous studies from Europe (*4*), the United States (*5**,**6*), and Japan (*7**,**8*). A representative US study that analyzed Medicare beneficiaries during 1997–2007 revealed that the prevalence of NTM disease increased from 20 to 47 cases/100,000 population and the prevalence was higher among women (*4*). A study conducted in Germany reported an increasing prevalence of NTM disease, from 2.3 cases/100,000 population in 2009 to 3.3 cases/100,000 population in 2014 (*4*). Studies in Japan, which is geographically close to South Korea, showed similar trends, but the rates were higher than those in the United States or Europe. The estimated annual prevalence of NTM disease in Japan in 2005 was 33–65 cases/100,000 population (*7*), and the incidence in 2015 was 14.7 cases/100,000 population (*8*), which were similar to our data. These results suggest that NTM infection is increasing globally, especially in East Asia, and that epidemiologic distribution can be affected by region or ethnicity. In particular, some studies have indicated that people in East Asia are vulnerable to NTM infection (*9**,**10*).

Another notable finding in our study was the difference in NTM prevalence by administrative division, which indicates that NTM infection is related to population density and the degree of urbanization. Supporting our data, other studies have attributed NTM infection to environmental exposure (*9*), and recent studies in the United States revealed that areas with high risk for NTM infection had higher population densities or higher education and income levels (*9**,**11*). In South Korea, the population density is highest in metropolitan cities. These data suggest the importance of epidemiologic surveillance in understanding NTM infection.

We frequently observed airway diseases in NTM infection in our study. A US study also reported COPD in 41% and bronchiectasis in 37% of NTM patients (*10*), whereas a study in Germany noted COPD or emphysema in 62%–79% of patients (*4*). One study reported mental disorders as concurrent NTM-related conditions (*12*). In our study, depression was also identified in NTM, which might be explained by the burden of COPD (*13*). These data suggest that an assessment of underlying disease is needed in managing NTM infection.

Our study had some limitations. First, because we used diagnosis codes, the distinction between pulmonary and other diseases is unclear, and we could not confirm whether the patients completely met the diagnostic criteria of NTM infection. Second, because we only have data for 2007–2016, we could not exclude patients who had received diagnoses before 2007 when estimating the annual incidence rate. Finally, our definition of NTM infection on the basis of the International Classification of Diseases code may have underestimated the true disease prevalence (*14**,**15*) because prevalence could be affected by loss to follow-up or death during the study period.

In conclusion, we report a substantial increasing trend in the prevalence and incidence of NTM infection in South Korea during 2007–2016 and evaluated regional variations. These data facilitate a better understanding of the epidemiologic trends of NTM infection globally.

Appendix Additional information about nontuberculous mycobacterial infection in South Korea.
