# Supplementary material for: Epidemiology of Nontuberculous Mycobacterial Infection, South Korea, 2007–2016
Source: Emerg Infect Dis. 2019 Mar;25(3):569–72. doi: 10.3201/eid2503.181597 (PMC6390769; doi:10.3201/eid2503.181597)
Supplement: Appendix — Additional information about nontuberculous mycobacterial infection in South Korea. [file 18-1597-Techapp-s1.pdf]

Article DOI: https://doi.org/10.3201/eid2503.181597

Epidemiology of Nontuberculous Mycobacterial Infection, South Korea, 2007–2016

Appendix

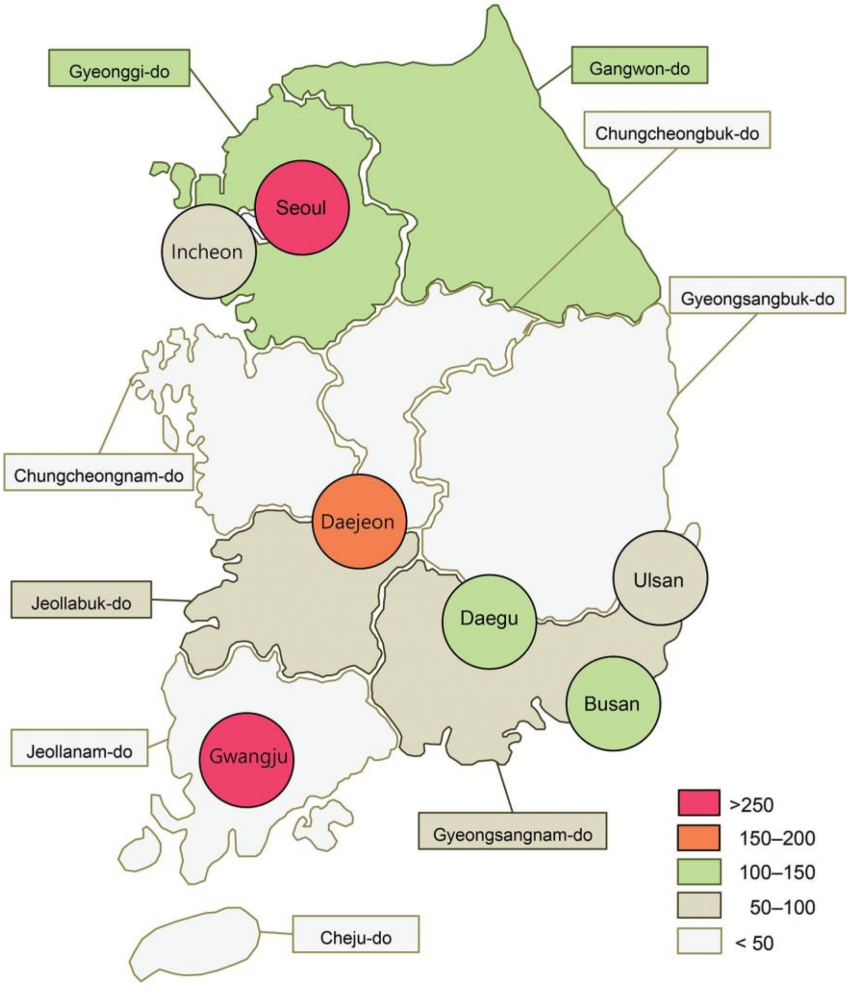

Appendix Figure. Overall period prevalence of nontuberculous mycobacterial infection by administrative division, adjusted for age and sex, South Korea, 2007–2016 (prevalence, no. cases/100,000 population).
